# Fusion of High Resolution Multispectral Imagery in Vulnerable Coastal and Land Ecosystems

**DOI:** 10.3390/s17020228

**Published:** 2017-01-25

**Authors:** Edurne Ibarrola-Ulzurrun, Consuelo Gonzalo-Martin, Javier Marcello-Ruiz, Angel Garcia-Pedrero, Dionisio Rodriguez-Esparragon

**Affiliations:** 1 Instituto de Oceanografía y Cambio Global, IOCAG, Universidad Las Palmas de Gran Canaria (ULPGC), Parque Científico Tecnológico Marino de Taliarte , 35214 Telde, Spain; javier.marcello@ulpgc.es (J.M.-R.); dionisio.rodriguez@ulpgc.es (D.R.-E.); 2 Centro de Tecnología Biomédica, Universidad Politécnica de Madrid (UPM), Campus de Montegancedo, 28223 Pozuelo de Alarcón, Spain; chelo@fi.upm.es (C.G.-M.); am.garcia@alumnos.upm.es (A.G.-P.)

**Keywords:** pansharpening, high resolution satellite images, WorldView-2, ecosystem management, classification

## Abstract

Ecosystems provide a wide variety of useful resources that enhance human welfare, but these resources are declining due to climate change and anthropogenic pressure. In this work, three vulnerable ecosystems, including shrublands, coastal areas with dunes systems and areas of shallow water, are studied. As far as these resources’ reduction is concerned, remote sensing and image processing techniques could contribute to the management of these natural resources in a practical and cost-effective way, although some improvements are needed for obtaining a higher quality of the information available. An important quality improvement is the fusion at the pixel level. Hence, the objective of this work is to assess which pansharpening technique provides the best fused image for the different types of ecosystems. After a preliminary evaluation of twelve classic and novel fusion algorithms, a total of four pansharpening algorithms was analyzed using six quality indices. The quality assessment was implemented not only for the whole set of multispectral bands, but also for the subset of spectral bands covered by the wavelength range of the panchromatic image and outside of it. A better quality result is observed in the fused image using only the bands covered by the panchromatic band range. It is important to highlight the use of these techniques not only in land and urban areas, but a novel analysis in areas of shallow water ecosystems. Although the algorithms do not show a high difference in land and coastal areas, coastal ecosystems require simpler algorithms, such as fast intensity hue saturation, whereas more heterogeneous ecosystems need advanced algorithms, as weighted wavelet ‘*à trous*’ through fractal dimension maps for shrublands and mixed ecosystems. Moreover, quality map analysis was carried out in order to study the fusion result in each band at the local level. Finally, to demonstrate the performance of these pansharpening techniques, advanced Object-Based (OBIA) support vector machine classification was applied, and a thematic map for the shrubland ecosystem was obtained, which corroborates wavelet ‘*à trous*’ through fractal dimension maps as the best fusion algorithm for this ecosystem.

## 1. Introduction

Natural ecosystems provide a wide variety of useful resources that enhance human welfare. However, in recent decades, there has been a decline in these ecosystem services, as well as their biodiversity [1]. In particular, coastal ecosystems are the most complex, dynamic and productive systems in the world [2]. This creates a demand to preserve environmental resources; hence, it is of great importance to develop reliable methodologies, applied to new high resolution satellite imagery. Thus, the analysis, conservation and management of these environments could be studied, in a continuous, reliable and economic way, and at the suitable spatial, spectral and temporal resolution. However, some processing tasks need to be improved; for instance, the weaknesses in the classification and analysis of land and coastal ecosystems on the basis of remote sensing data, as well as the lack of reliability of the maps; particularly, the extreme difficulty to monitor the coastal ecosystems from remote sensing imagery due to the low reflectivity of these areas covered by water.

The framework in which the study has been developed is the analysis of both coastal and land ecosystems through very high resolution (VHR) remote sensing imagery in order to obtain high quality products that will allow the comprehensive analysis of natural resources. In this context, remote sensing imagery offers practical and cost-effective means for a good environmental management, especially when large areas have to be monitored [3] or periodic information is needed. Most VHR optical sensors provide a multispectral image (MS) and a panchromatic image (PAN), which require a number of corrections and enhancements. Image fusion or pansharpening algorithms are important for improving the spatial quality of information available. The pansharpening data fusion technique could be defined as the process of merging MS and PAN images to create new multispectral images with a high spatial resolution [4,5]. This fusion stage is important in the analysis of such vulnerable ecosystems, mainly characterized by heterogeneous and mixed vegetation shrubs, with small shrubs in the case of terrestrial ecosystems and the complexity of seagrass meadows or algae distribution in shallow water ecosystems.

Image fusion techniques combine sensor data from different sources with the aim of providing more detailed and reliable information. The extensive research into image fusion techniques in remote sensing started in the 1980s [6,7]. Generally, image fusion can be categorized into three levels: pixel level, feature level and knowledge or decision level [8,9], and pansharpening is performed at the pixel level.

Many pansharpening techniques have appeared in recent decades, as a consequence of the launch of very high resolution sensors [10,11,12,13,14]. An ideal pansharpening algorithm should have two main attributes: (i) enhancing high spatial resolution; and (ii) reducing spectral distortion [15]. The simplest pansharpening methods, at the conceptual and computational level, are intensity-hue-saturation (IHS), principal component analysis (PCA) and Brovey transforms (BT). However, these techniques have problems because the radiometry on the spectral channels is distorted after fusion. New approaches, such as wavelet transformations and high pass filtering (HPF) [4,8,16,17,18], have been proposed to address particular problems with the traditional techniques.

On the other hand, quality evaluation is a fundamental issue to benchmark and optimize different pansharpening algorithms [18,19], as there is the necessity to assess the spectral and spatial quality of the fused images. There are two types of evaluation approaches commonly used: (1) qualitative analysis (visual assessment); and (2) quantitative analysis (quality indices). Visual analysis is a powerful tool for capturing the geometrical aspect [20] and the main color disturbances. According to [10], some visual parameters are necessary for testing the properties of the image, such as the spectral preservation of features, multispectral synthesis in fused images and the synthesis of images close to actual images at high resolution. On the other hand, quality indices measure the spectral and the spatial distortion produced due to the pansharpening process by comparing each fused image to the reference MS or PAN image. The work in [21] categorized them into three main groups: (i) spectral quality indices such as the spectral angle mapper (SAM) [22] and the spectral relative dimensionless global error, in French ‘*erreur relative globale adimensionnelle de synthése*’ (ERGAS) [23]; (ii) spatial quality indices, i.e., the spatial ERGAS [24], the frequency comparison index (FC) [25] and the Zhou index [26]; and (iii) global quality indices, such as the 8-band image quality index (Q8) [27]. On the other hand, there are several evaluation techniques with no reference requirement, such as the quality with no reference (QNR) approach [28].

In this study, the main goal is to assess which pansharpening technique, using Worldview-2 VHR imagery with eight MS bands, provides a better fused image. Future research will be focused on the classification of the vulnerable ecosystems, in order to obtain specific products for the management of coastal and land resources, in contrast to several studies assessing and reviewing pansharpening techniques [11,16,20,29,30,31,32,33,34]. Further specific goals in this paper are: (i) the study of the spatial and spectral quality of pansharpened bands covered by and outside the PAN wavelength range; (ii) analysis of pansharpening algorithms’ behavior in vulnerable natural ecosystems, unlike the majority of previous studies, which apply the pansharpening techniques in urban areas or on other land cover types; and (iii) novel assessment in VHR image fusion in shallow coastal waters, whilst being aware of the pansharpening difficulty of these ecosystems. Although other authors apply fusion in water areas, such as [35,36], they use Landsat imagery, not VHR imagery. The aim of the last point is to identify which techniques were more suitable for shallow water areas and the improvement achieved. This information can lead to obtaining more accurate seafloor segmentation or mapping of coastal zones [37]; hence, studies on the state of conservation of natural resources could be conducted.

Finally, in order to strengthen the study, a final thematic map of the shrubland area was carried out. Thus, it will analyze the influence of the fusion on the classification results which serve to obtain accurate information for the conservation of natural resources. This study can be found in more detail in [38].

The paper is structured as follows: Section 2 includes the description of the study area, datasets, the image fusion methods used in the analysis and the evaluation methodology. The visual and quantitative evaluation of the different algorithms, as well as map analysis are presented in Section 3. Section 4 includes the critical analysis of the results. Finally, Section 4 summarizes the main outcomes and contributions.

## 2. Materials and Methods

### 2.1. Study Area

**Figure 1:**
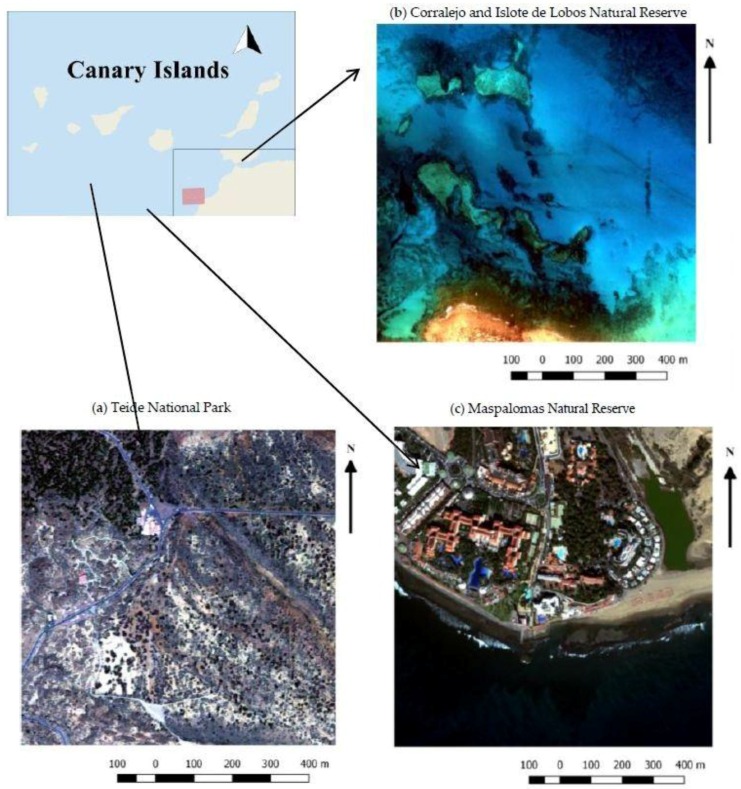
Study areas from the Canary Islands: (**a**) Teide National Park; (**b**) Corralejo and Islote de Lobos Natural Park; and (**c**) Maspalomas Natural Reserve.

This study focuses on three types of vulnerable ecosystems found in different islands of the Macaronesia region. Macaronesia is considered a geological and a biodiversity hotspot due to the volcanic origin and due to the high degree of vulnerability that insular ecosystems are subjected to, mainly as a consequence of climate change and anthropogenic pressure. The ecosystems selected from the Canary Islands were: the shrubland ecosystem, the coastal ecosystem and, finally, a mixed ecosystem surrounded by a touristic area and a lagoon, as a transitional system within the coastal and land ecosystems. The Canary Islands are one of the most remarkable biodiversity areas on the planet [39], and they are chosen as a representative sample of these ecosystems because of the availability of VHR remote sensing imagery and simultaneous field data. Figure 1 displays the 3 protected areas considered in the analysis.

As regards shrubland ecosystems, it is important to highlight the large concentration of vascular plants, which are highly vulnerable to environmental changes. In coastal areas, an intensive natural fragility also appears due to the interaction of a great variety of environmental factors. Moreover, the coastal occupation of urban areas and the development of tourism increase this frailty and vulnerability. In particular, dune systems are affected by this urban-touristic expansion [40,41,42,43]. Furthermore, many coastal ecosystems contain seagrass meadows [44]. The importance of these particular meadows is related to the ocean productivity as they are one of the most valuable ecosystems in the world. In addition, these meadows are part of the solution to climate change, not only producing oxygen, but storing up to twice as much carbon per unit area as the world’s temperate and tropical forests [45].

As indicated, three sensible and heterogenic protected areas of the Canary Islands have been selected (Figure 1) as representative examples of more general ecosystems: the Teide National Park (Tenerife Island), as an example of shrubland ecosystems, the Corralejo Natural Park and Islote de Lobos (Fuerteventura), representing coastal ecosystems, and the Maspalomas Natural Reserve (Gran Canaria Island), an important coastal-dune ecosystem with significant tourism pressure, called the mixed ecosystem in this paper, not only because it is the transitional region within the coastal and land ecosystem, but also due to the inner water ecosystem, known as the Maspalomas lagoon.

Other similar shrubland ecosystems can be identified around the world, for example: Pico do Pico in the Azores, Mt. Halla in South Korea and Hawaii or the Galapagos. Corralejo and Maspalomas are important coastal sand dune systems in Europe, as they contain a large degree of biodiversity [46]. The importance of studying these ecosystems is their similarity with other Mediterranean, temperate or tropical parts of the world: Tuscany (Italy), Doñana National Park (Spain), as well as other parts of the world, such as Parangtritis (Java, Indonesia), Malaysia, Philippines, Vietnam, NE Queensland, the tropical coast of Brazil, the West African coast, Cuba, the Galapagos islands, the West Indies, Cox’s Bazaar (Bangladesh), Hawaii, Ghana, the coast of India or Christmas Island [47].

### 2.2. Datasets

**Figure 2:**
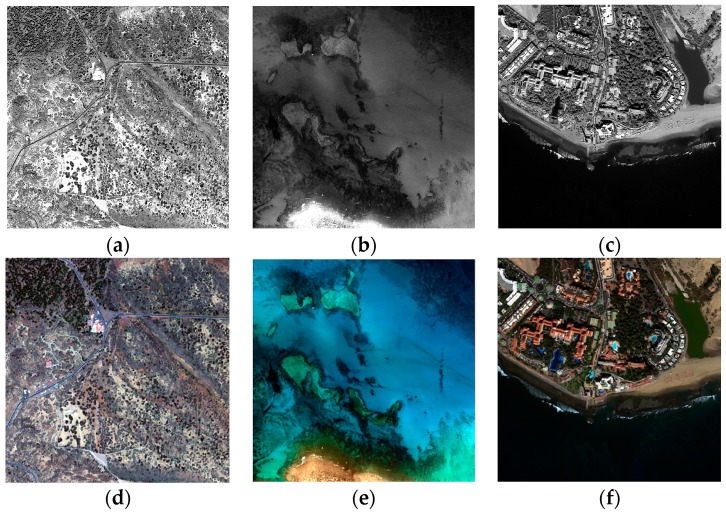
PAN and MS scenes of WorldView-2 images (512 × 512 pixels for the MS image): (**a**,**d**) shrub land ecosystem; (**b**,**e**) coastal ecosystem; (**c**,**f**) mixed ecosystem with urban area and inner water lagoon.

The WorldView-2 (WV-2) satellite, launched by Digital Globe on 8 October 2009, was the first commercial satellite to provide a VHR sensor with one PAN and eight MS bands (Table 1). WV-2 ortho-ready imagery of the three representative ecosystems were used in the study. Images of the Canary Islands and the central locations of the corresponding three study areas are detailed in Table 2. In order to reduce the computation times in the multiple analyses, 512 × 512 MS pixel scenes were used. Figure 2 displays the PAN band and the corresponding resized MS image (RGB composite). They were selected for their spectral and spatial richness and the content of land and coastal areas. In addition, the scenes include different coverages, basically predominating coastal areas, shallow waters, vegetation and urban regions, and they contain features with different shapes and edges.

### 2.3. Image Fusion Methodology

**Figure 3:**
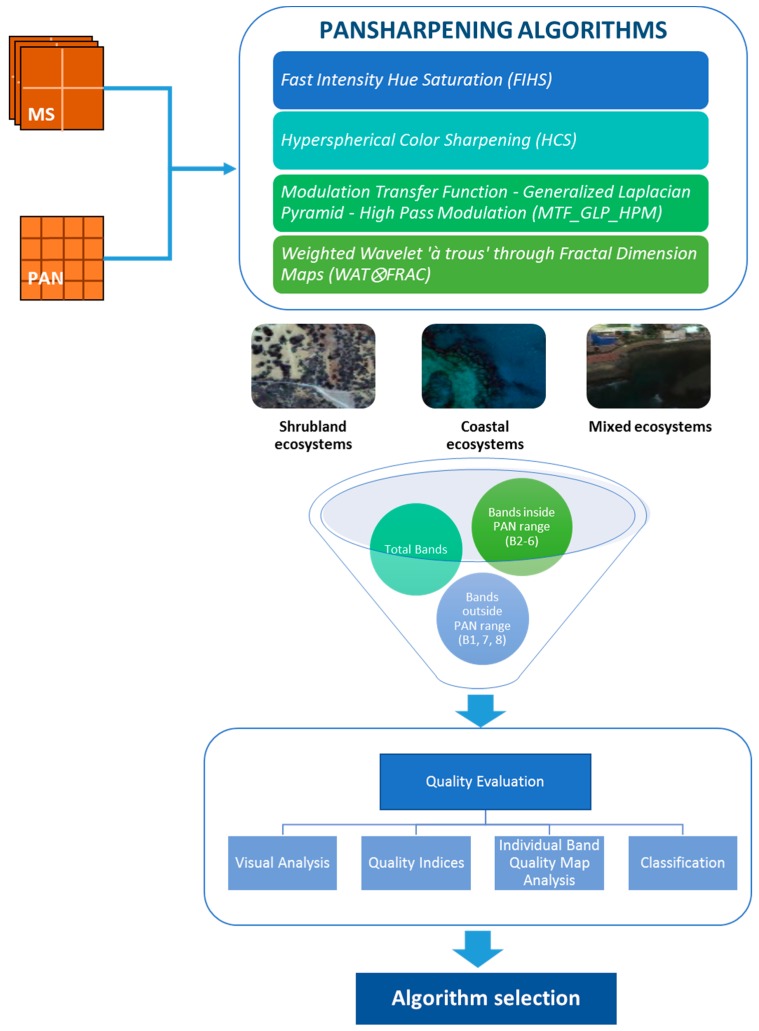
The flow diagram followed in the study for each scenario.

In the flow shown in Figure 3, every step of the methodology for assessing which algorithm gives the best fused image for each area is presented.

The first four pansharpening techniques were implemented in the three different vulnerable ecosystems; afterwards, a visual and quantitative assessment was undertaken in order to evaluate the pansharpening results in the different fused images. The quality assessment was carried out for the whole set of MS bands, as well as for those MS bands covering the range of the PAN channel (Bands 2–6) and those outside this range (Bands 1, 7 and 8). Furthermore, an individual band quality map analysis was carried out in the best fused images according to each ecosystem type. Finally, a classification map is obtained in the different fused images, to analyze the influence of the pansharpening in the classification result.

#### 2.3.1. Image Fusion Methods

After a review of the state-of-the-art in pansharpening techniques, at the pixel level, a preliminary assessment was carried out selecting classic and new algorithms that could achieve good performance with WV-2 imagery. Some algorithms specifically adapted to the WorldView-2 sensor have been chosen. An initial visual and quantitative assessment was carried out using a total of 12 different pansharpening techniques in these data, but only the best algorithms were selected for this study. The final election of these four algorithms was carried out with the same methodology explained in this paper. Thus, a visual and quantitative evaluation was performed to assess the spectral and the spatial quality of each algorithm, taking into account the compromise between both qualities in each fused image, depending on each ecosystem type. Thus, after obtaining an objective ranking of the 12 algorithms selected using the Borda count method, explained in Section 2.3.2, the final best four pansharpening algorithms were selected, in order to obtain the most suitable fused image for each ecosystem. Next, a brief overview of each family of the algorithms selected in the study is presented. Any formula or block diagram is omitted (for detailed information, see the references).

- Fast intensity hue saturation (FIHS) [48]: It uses the spectral bands to estimate the new component *I*. The spatial detail is extracted, computing the difference between the PAN band and the new component *I*. The spatial detail is injected into any number of bands.
- Hyperspherical color sharpening (HCS): This pansharpening algorithm is designed specifically for WV-2 by [49] based on the transformation between any native color space and the hyperspherical color space. Once transformed into HCS, the intensity can be scaled without changing the color, essential for the HCS pansharpening algorithm [15,50]. The transformation to HCS can be made from any native color space.
- Based on modulation transfer function: The modulation transfer function (MTF) is a function of the sensor spatial frequency response, describing the resolution of an imaging system [28]. Generalized Laplacian pyramid (GLP) is an extension of the Laplacian pyramid where a scale factor different from two is used [10]. Finally, in high pass modulation (HPM), the PAN image is multiplied by each band of the original MS image and normalized by a low pass filtered version of the PAN image in order to estimate the enhanced MS image bands.
- Weighted wavelet ‘*à trous*’ method through fractal dimension maps (WAT⊗FRAC) [14]: This method is based on the wavelet ‘*à trous*’ algorithm. A mechanism that controls the trade-off between the spatial and spectral quality by introducing a weighting factor (α_i_) for the PAN wavelet coefficients is established. However, this factor only discriminates between different spectral bands, but not between different land covers; therefore, the authors proposed a new approach [51], defining a different weight factor α_i_ (x, y) for each point of each band. α_i_ (x, y) was defined as a fractal dimension map (FDM) with the same size as the original image. A preliminary analysis was carried out using three different window sizes for the windowing process: 7, 15 and 27.

#### 2.3.2. Quality Evaluation Methodology

(a) Visual quality:

A visual analysis was the first step in the quality assessment. Through this approach, the main errors on an overall scale were observed, and then, local artefacts where analyzed closely. For the visual spectral assessment, fused true color images were compared to their original MS image, used as the reference, and its spectral features compared with the original MS image. Firstly, several aspects of the image features were taken into account in the spectral assessment, such as the tone, contrast, saturation, sharpness and texture. Furthermore, we paid attention to color disturbances. False color composites were produced in order to evaluate the fused NIR bands, where we focused on the same aspects as mentioned above. Finally, we concentrated on linear features, specific objects, surfaces or edges of buildings in order to observe spatial disturbances using the PAN as a reference.

(b) Quality indices

There is no current consensus in the literature on the best quality index for pansharpening images [52]. The quantitative quality evaluation of fused images is a debated issue since no reference image exists at the pansharpened resolution [4,20]. A number of statistical evaluation indices were used to measure the quality of the fused images. Each fused image is compared to the reference MS image.

The spectral quality assessment measures the spectral distortion brought by the pansharpening process. The metrics considered in the analysis are as follows:

- The spectral angle mapper (SAM) was designed to determine the spectral similarity in a multidimensional space [22] (Equation (1) in Table 3).
- The spectral ERGAS (relative dimensionless global error) is an overall quality index sensitive to mean shifting and dynamic range change (Table 2, Equation (3)). The $rmse_{i}$ (root mean square error) is calculated by its standard definition [23].

The correlation coefficient was not selected as spectral index due to its low capability in techniques with low quality differences.

On the other hand, the spatial detail information of each fused band is compared with the spatial information of the reference PAN image. The metrics considered in the analysis are as follows:

- The spatial ERGAS was proposed by [24]. It is a new spatial index considering the PAN band as a reference (Table 3, Equation (3)).
- The frequency comparison index (FC) is proposed by [25]. It is based on the discrete cosine transform (*dct*) for the spatial assessment (Table 3, Equation (4)).
- The Zhou index (Table 3, Equation (5)) measures the spatial quality computing correlation between the high pass filtered fused image ($FUS_{i}^{high\_pass}$) and PAN ($PAN^{high\_pass}$) image for each band.

Finally, as the global quality assessment:

- The Q8 index is a generalization to eight-band images of the Q index [27]. It is based on the different statistical properties of the fused and MS images (Table 3, Equation (6)).

In order to identify, in an objective way, the best fused image for each ecosystem, the best algorithms at the spectral, spatial and global level for each scene have been established by the Borda count rank aggregation method (Equation (7)) [53]. Consider *U* a set of items *i*, called the universe, and *R* a set of the rank list, where τ is an item of the rank list. The method is equivalent to: for each item *i* ∈ *U*, a rank list τ ∈R, and considering Borda normalized weight $\omega^{\tau }\left(i\right)$, the fused rank list $\hat{\tau }$ is ordered with respect to the Borda score $s^{\hat{\tau }}$, where the Borda score of an item *i* ∈ *U* in $\hat{\tau }$ is defined as:

(7) $$s^{\hat{\tau }}\left(i\right)=\;\underset{\tau \;\in R}{\sum }\omega^{\tau }\left(i\right)$$

### 2.4. Classification Maps

A supervised classification technique was applied only in the shrubland ecosystem scene in order to analyze the influence of the different pansharpening techniques in the generation of thematic maps [38]. The first step was to determine the classes appearing in the image and obtain a sufficient number of training samples. The classes chosen for this ecosystem were selected by experts of the Teide National Park, the vegetation classes being: *Spartocytisus supranubius* (Teide broom), *Pterocephalus lasiospermus* (rosalillo de cumbre), *Descurainia bourgaeana* (hierba pajonera) and *Pinus canariensis* (Canarian pine). Urban, road and bare soil classes were also included. In the second step, the OBIA process starts with a segmentation of the input images into local groups of pixels, i.e., objects, that become spatial units in the later analysis, classifications and accuracy assessment. Object shape, size and spectral properties depend on both the segmentation approach and the research goals. The image was segmented using the multiresolution segmentation followed by the spectral difference segmentation, in order to preserve the small objects of interest to classify. Once the objects are obtained from the segmentation techniques, classification algorithms can be applied. The last step was to determine the classification algorithm; in our case, we applied the novel object-based or OBIA classification approach [54], using support vector machine (SVM) as the supervised classifier [55]. SVM is a related supervised learning method that analyzes data and recognizes patterns, used for classification and regression analysis. The standard SVM takes a set of input data and predicts, for each given input, which of the different possible classes the input is a member. Given a set of training examples, each marked as belonging to the categories, an SVM training algorithm builds a model that assigns new examples into one category [56]. Thematic maps were obtained after implementing the SVM classifier in each fused image. Afterwards, the accuracy of the classification must be measured; in this case, testing samples were collected. The statistical accuracy assessment technique used was the overall accuracy and the kappa coefficient.

## 3. Results

This section is divided into three main blocks: (i) the visual assessment; (ii) the quantitative evaluation based on the quality indexes; and (iii) the thematic maps resulting from the OBIA classification in the shrubland ecosystem.

### 3.1. Visual Evaluation

**Figure 4:**
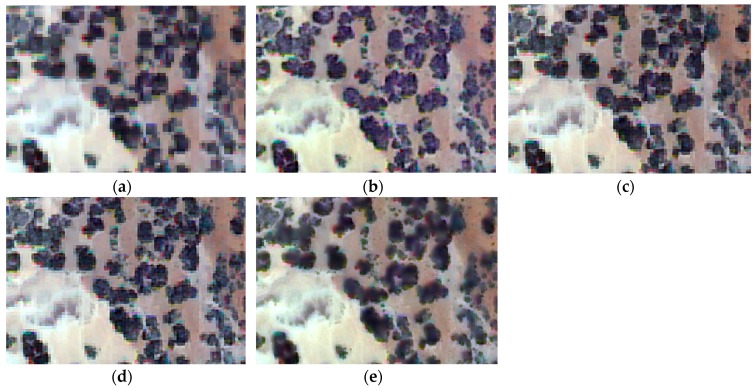
True color fused images of the shrubland region: (**a**) original MS; (**b**) FIHS; (**c**) HCS; (**d**) MTF_GLP_HPM; (**e**) WAT⊗FRAC with a window size of seven.

**Figure 5:**
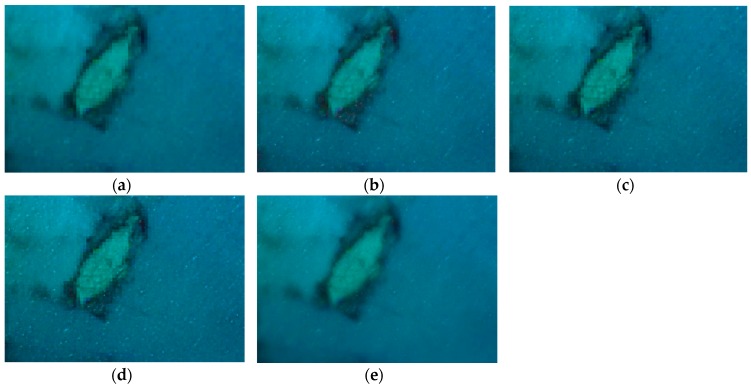
True color fused images of the coastal region: (**a**) original MS; (**b**) FIHS; (**c**) HCS; (**d**) MTF_GLP_HPM; (**e**) WAT⊗FRAC with a window size of 27.

**Figure 6:**
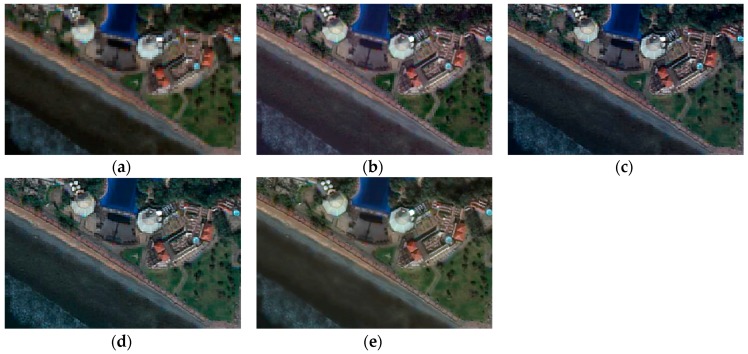
True color fused images of the mixed ecosystem with an urban region: (**a**) original MS; (**b**) FIHS; (**c**) HCS; (**d**) MTF_GLP_HPM; (**e**) WAT⊗FRAC with a window size of 15.

For the visual analysis, both color and edge preservation are the most important criteria to evaluate the performance of image fusion techniques in order to identify the fusion technique that provides the fused image with the highest spectral and spatial quality. To facilitate the visual inspection and for a more detailed spatial analysis, a zoom of the previous scenes is shown in Figure 4, Figure 5 and Figure 6. It is important to highlight that, after the preliminary assessment, robust pansharpening algorithms have been selected, so all fusion results are satisfactory, and the spectral differences are difficult to appreciate visually, except in some specific areas. We want to underline that, to the best of our knowledge, this is the first time pansharpening algorithms have been assessed in coastal ecosystems using VHR imagery. This improvement in the spatial quality due to the application of fusion techniques could be useful to improve seafloor or benthic classification of shallow waters (i.e., coral reefs or seagrass meadows).

The visual interpretation at the spectral level in the shrubland region (Figure 4) indicates that every algorithm, except for WAT⊗FRAC, produces a slight color distortion over the entire fused image. On the other hand, in the preliminary analysis, WAT⊗FRAC with a window size of seven pixels provides the best fused image from among the WAT⊗FRAC algorithms, although the differences are minimal.

Observing the coastal region (Figure 5), differences among the techniques are basically undetectable at the spectral level. The WAT⊗FRAC window size, which gives a slightly better result in this image, is 27.

Visual results similar to those of the shrubland region appear in the mixed ecosystem (Figure 6), where the FIHS, HCS and MTF algorithms show a more perceptible color distortion in the sea than in buildings. The WAT⊗FRAC window size chosen for this scene is 15.

Spatially, the differences are clearer than spectrally. In the case of the HCS and MTF_GLP_HPM techniques, although they maintain the spectral information well, the spatial details are not satisfactorily injected, thus not achieving a good spatial enhancement. Furthermore, the blurred aspect found in the WAT⊗FRAC algorithm is because the algorithm makes uniform the homogenous areas found in the multispectral image, which appear as heterogeneous areas in the panchromatic image. Thus, the ‘salt and pepper’ effect is avoided with it, obtaining a better classification and more accurate thematic maps, as demonstrated in Section 3.3.

**Figure 7:**
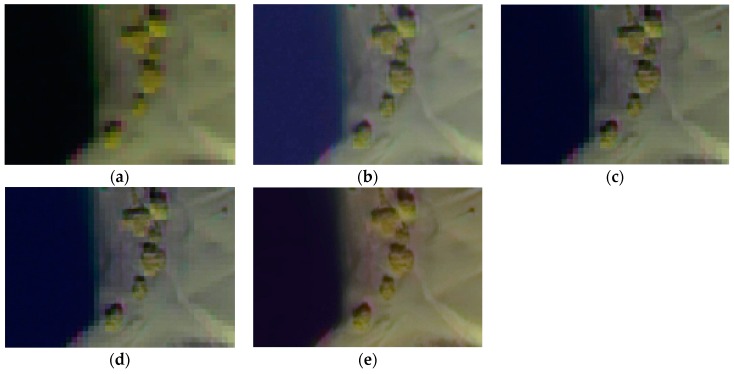
False color fused images using Bands 8, 7 and 1 color composition (bands outside the PAN range): (**a**) original MS; (**b**) FIHS; (**c**) HCS; (**d**) MTF_GLP_HPM; (**e**) WAT⊗FRAC.

Finally, Figure 7 shows an example of the false color composite using bands outside the PAN range (Bands (B) 8, 7 and 1).

In this case, a region with soil, vegetation and water is chosen in order to analyze the behavior of the different techniques. Spectrally, algorithms show a more significant color distortion in the water area. At the spatial level, pansharpening algorithms demonstrate the same behavior as in the true color composite, with WAT⊗FRAC achieving an optimum result.

### 3.2. Quantitative Evaluation of Pansharpened Images

As was mentioned in Section 2.4, in order to perform an objective evaluation of the pansharpening techniques, six spectral and spatial quality indices have been computed on the complete scenes of Figure 2. First, quantitative indices were calculated for all of the bands in the MS image in the three ecosystems. Second, the pansharpening performance for bands covered by and outside of the PAN range was assessed. Finally, an individual band quality map analysis was carried out (Figure 3).

#### 3.2.1. Shrubland Ecosystems

The quality analysis of all multispectral bands can be appreciated in Table 4. SAM, spectral ERGAS and Q8 confirm that the MTF method and the HCS algorithm provide better spectral performance, while FIHS and WAT⊗FRAC get the lowest result, even though there is not a large difference between the highest and the lowest value. As regards the spatial performance, WAT⊗FRAC is confirmed as the best spatial quality method, while HCS shows the worst values. Furthermore, these results confirm the compromise between the spectral and spatial quality, in which the best fused image considered in this study would be the one that provides the best compromise between them. Hence, the Borda count method is used a posteriori to observe this compromise.

The Borda count method has allowed analyzing the balance between the spectral and spatial quality in the pansharpening algorithms to be distinguished to avoid any bias in the global result (Table 4). The results obtained by the Borda count method including the respective spectral and spatial quality indices appear in the ‘spectral’ and ‘spatial’ columns. On the other hand, the ‘global’ column shows the results using the Borda count method in every algorithm considering all of the quality indices.

Analyzing Table 4, WAT⊗FRAC generates the best fused image, not only in the overall evaluation, but also at the spatial level.

**Figure 8:**
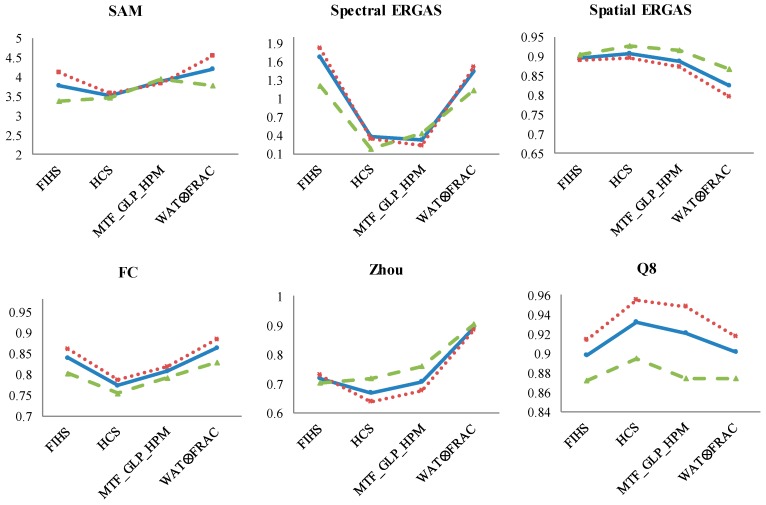
Results of the quality indices for the shrubland ecosystem fused image considering the three different MS band combinations (blue: total bands; red: Bands 2–6; green: Bands 1, 7 and 8). X-axis: panharpening algorithms and Y-axis: range values of each quality indices (SAM: better value closer to 0; Spectral and Spatial ERGAS: better values closer to 0; FC: values between 0 and 1, better closer to 1; Zhou: values between 0 and 1, better closer to 1; Q8: values between 0 and 1, better closer to 1).

As regards the band analysis based on the PAN range, the behavior of the quality index is analyzed with respect to the MS fused bands. Thus, Figure 8 shows the quantitative values obtained for the pansharpened bands within the PAN spectral range (B 2–6) and outside of this range (B 1, 7 and 8). The results for the complete set of bands are included as a reference.

SAM and spectral ERGAS indices provide similar results, where fused bands outside of the PAN range have better quality than the bands within the PAN range, the results for the complete set of bands being an average of them. There is an exception with MTF_GLP_HPM, providing similar results irrespective of the bands that are used for their estimation. While the analysis of the spatial indices, in general, shows that bands inside the PAN range achieve better spatial quality.

#### 3.2.2. Coastal Ecosystems

Table 5 includes the values of the quality indices for all bands obtained when applying the different fusion methods in the coastal ecosystem scenario. It is important to point out that most of the scene is covered by shallow water. The SAM index shows as the best fused images the ones obtained by MTF_GLP_HPM and FIHS; however, it does not demonstrate a large variability in the results, just like that of Q8. In the case of spectral ERGAS, MTF_GLP_HPM shows the best fused image. Spatial indices also show some variability, where spatial ERGAS and FC identify FIHS as the best method, while the WAT⊗FRAC algorithm gets the best quality result in Zhou. All spatial indices agree that the lowest quality image is achieved by the MTF_GLP_HPM and HCS methods.

According to the Borda count method, the best algorithm for getting a good fusion in coastal ecosystems is FIHS, which achieves a good balance between the spectral and the spatial quality.

**Figure 9:**
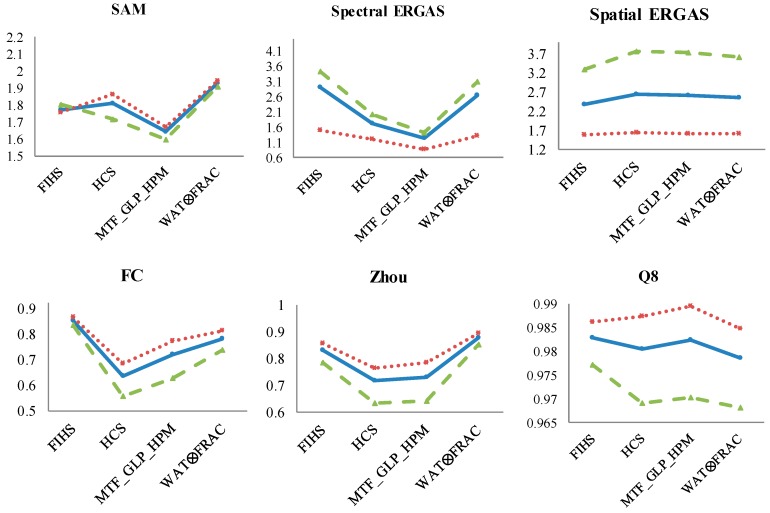
Results of the quality indices for the coastal ecosystem fused image considering the three different MS band combinations (blue: total bands; red: Bands 2–6; green: Bands 1, 7 and 8). X-axis: panharpening algorithms and Y-axis: range values of each quality indices (SAM: better value closer to 0; Spectral and Spatial ERGAS: better values closer to 0; FC: values between 0 and 1, better closer to 1; Zhou: values between 0 and 1, better closer to 1; Q8: values between 0 and 1, better closer to 1).

With respect to the band analysis based on the PAN range (Figure 9), from the spectral point of view, SAM provides similar values between the fusion algorithms, and the spectral ERGAS shows better quality images for the MS bands covered by the PAN range, as expected, except in FIHS. Concerning the spatial indices, the Spatial ERGAS and FC metrics achieve a superior spatial quality for Bands 2–6, while Zhou shows very similar results. The Q8 index also gives higher values to the bands covered by the PAN.

#### 3.2.3. Mixed Ecosystems

The quantitative evaluation in all bands for the mixed ecosystem is shown in Table 6. According to the spectral and spatial quality indices, the results are similar to those of shrubland ecosystems. The MTF_GLP_HPM technique provides the best result at the spectral level, while the WAT⊗FRAC algorithm does so at the spatial level.

**Figure 10:**
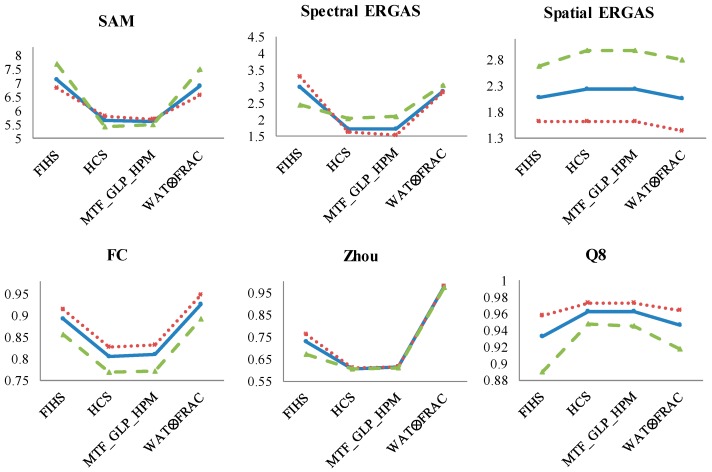
Results of the quality indices for the mixed ecosystem fused image considering the three different MS band combinations (blue: total bands; red: Bands 2–6; green: Bands 1, 7 and 8). X-axis: panharpening algorithms and Y-axis: range values of each quality indices (SAM: better value closer to 0; Spectral and Spatial ERGAS: better values closer to 0; FC: values between 0 and 1, better closer to 1; Zhou: values between 0 and 1, better closer to 1; Q8: values between 0 and 1, better closer to 1).

Looking at the spectral quality indices in the mixed ecosystem scene (Figure 10), the same behavior is found for the Spectral ERGAS, which obtains the worst fused image using MS bands outside of the PAN range for the fusion, while in SAM, the values are, in general, very similar between them. With respect to the spatial quality, the bands inside the PAN range obtain better quality values. Finally, Q8 obtains worse quality results for the MS bands outside of the PAN range, as expected.

In this case, the WAT⊗FRAC algorithm also achieves the best score in the Borda count rank, making it the most reliable algorithm for this kind of ecosystem.

#### 3.2.4. Individual Band Quality Map Analysis

Quality values for each individual band in each scene are included in Table 7. The Q8 overall index does not show the variability at the local level; thus, quality map analyses were carried out using the Q8 metric with a block size of 64 in order to examine the quality at local level in each band using the best algorithms for each area. The maximum quality index is achieved if a window is not used. This block size of 64 was chosen because increasing the block size increases the index values, and thus, the values obtained in the quality maps could be comparable to the values obtained for the overall Q8. In each quality map, a blue scale has been used with white indicating a higher similarity (Q8 metric) between the original and the fused MS band.

**Figure 11:**
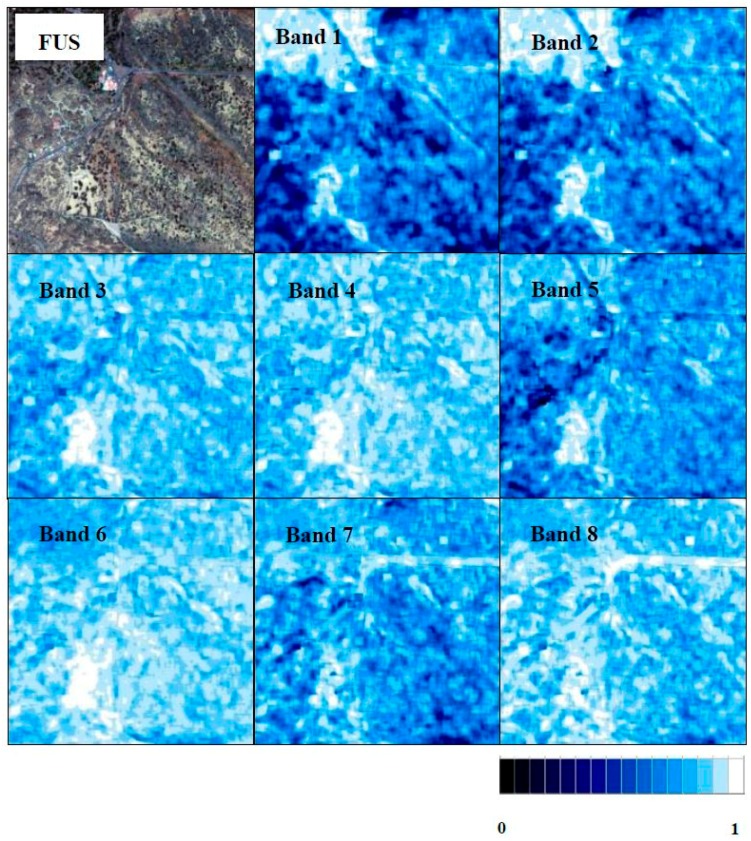
Fused image with the WAT⊗FRAC of the shrubland ecosystem and its quality maps for each band (scale from 0–1, zero being less fusion quality and one the highest fusion quality).

As indicated, the WAT⊗FRAC algorithm was selected as the best compromise to fuse images in a shrubland ecosystem scenario. Quality maps of WAT⊗FRAC (window size of seven) are presented in Figure 11. Better results can be appreciated for Bands 3–8, with Band 4 achieving the best fusion. On the other hand, Bands 1 and 2 (coastal blue and blue) do not show good quality in some areas, with a greater concentration of dark blue pixels, in accordance with the lowest quality results of Table 7.

**Figure 12:**
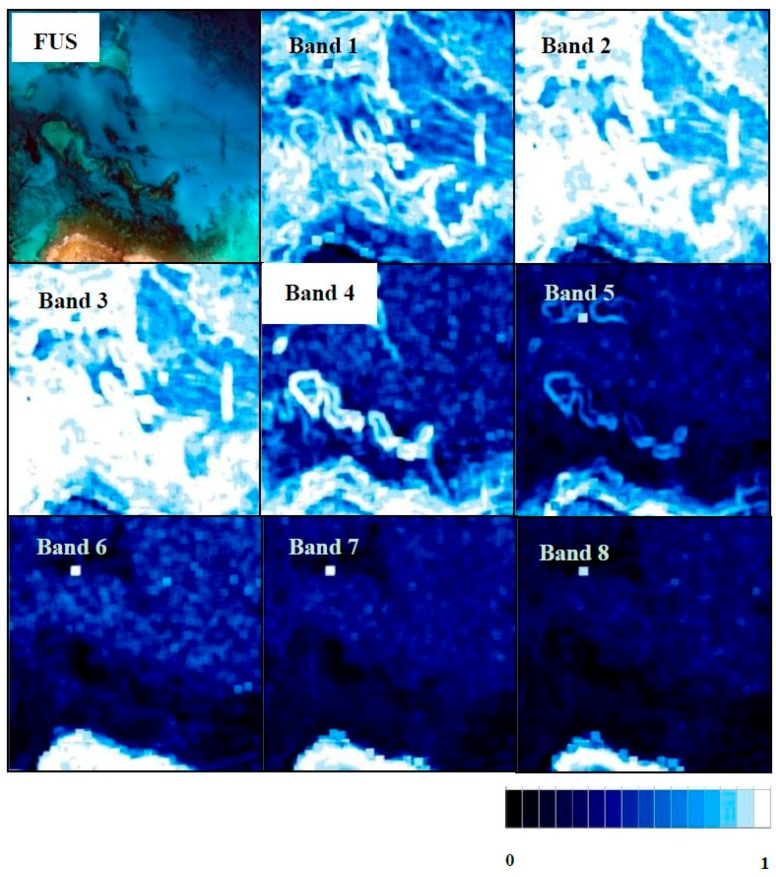
Fused image with FIHS of the coastal ecosystem and its quality maps for each band (scale from 0–1, zero being less fusion quality and one the higher fusion quality).

As regards the coastal ecosystem, in the individual band quality maps of FIHS (Table 7 and Figure 12), the higher quality is achieved in Bands 2 and 3, with values over 0.88. For Band 1 (0.764), a light blue aspect (medium quality) in the sea area is clear, whereas, in the land area, both bands get dark blue pixels in the quality maps with this algorithm. From Bands 4–8, the quality increases in land areas, showing mostly dark blue pixels; however, the quality in sea areas decreases considerably. Band 8 gets a quality value of 0.239, while Bands 5–7 show values around 0.3–0.4. Regardless of the fact that the FIHS algorithm gets the best fusion for this scenario, the quality maps are not very satisfactory for longer wavelengths, but this portion of the spectrum is of minimum interest in seafloor mapping applications due to the low capability of light to penetrate the water.

**Figure 13:**
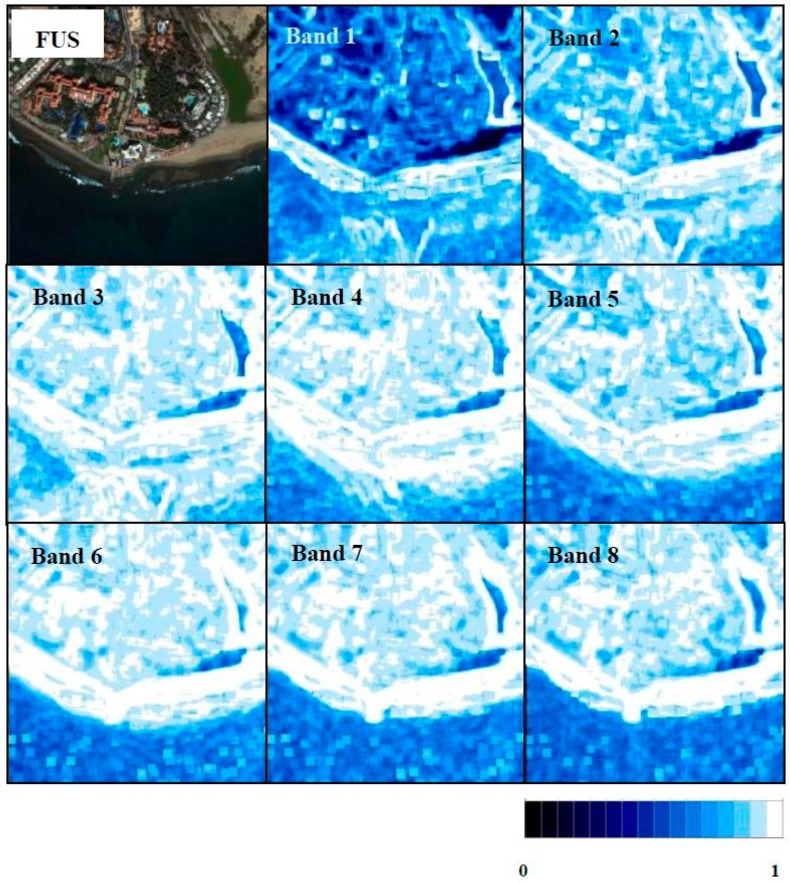
Fused image with WAT⊗FRAC of the mixed ecosystem and its quality maps for each band. Scale from 0–1, zero being less fusion quality and one the higher fusion quality.

Concerning the quality maps of the WAT⊗FRAC algorithm in mixed ecosystems (Figure 13), they present a similar behavior to that of shrubland areas. Specifically, Bands 3 and 4 have the highest quality values (0.905 and 0.890, respectively, as presented in Table 7), while Bands 1 and 2 show lower quality (0.695 for Band 1 and 0.842 for Band 2). On the other hand, water areas have, in general, worse quality as the band number increases.

### 3.3. Thematic Maps of Shrubland Ecosystems

Table 8 shows the overall accuracy and the kappa coefficient for the SVM object-based classification applied to each fused image. The best result is obtained in the WAT⊗FRAC fused image, corroborating the results achieved in the Quantitative Evaluation section, where WAT⊗FRAC shows the best fusion result.

**Figure 14:**
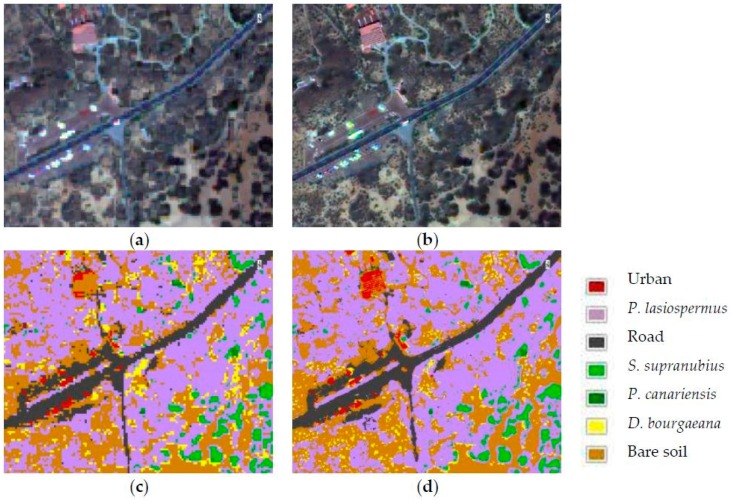
Zoom of the original MS (**a**) and WAT⊗FRAC fused image (**b**) of the thematic maps obtained by the OBIA-SVM classifier applied to the multispectral image (**c**) and WAT⊗FRAC fused image (**d**) in the shrubland ecosystem.

Finally, Figure 14 presents the thematic map from the shrubland ecosystem obtained in the original MS image and in the WAT⊗FRAC fused image by SVM classification. It is observed how the fusion is a fundamental preprocessing in these complex ecosystems because some classes of interest are not well delimited in the original multispectral thematic maps. On the other hand, buildings (red) are erroneously classified as bare soil (brown) in the original multispectral image, and road limits (grey) and vegetation contours seem to be stepped, due to the pixel size in the original image. The results were analyzed by the experts of the national park, confirming a good agreement with respect the real vegetation of the area.

## 4. Conclusions

As indicated, the main objective of this work was to select the pansharpening algorithm that provides the image with the best spatial and spectral quality for land and coastal ecosystems. Due to this reason, the most efficient pansharpening techniques developed in recent years have been applied, in order to achieve the highest spatial resolution of the MS bands while preserving the original spectral information, and assessed. As not a single pansharpening algorithm has exhibited a superior performance, the best techniques have been evaluated in three different types of ecosystems, i.e., heterogenic shrubland ecosystems, coastal systems and coastal-land transitional zones with inland water and an urban area. Fusion methods have frequently been applied to land and urban areas; however, a novel analysis has been conducted covering their evaluation in areas of shallow water using VHR imagery, as well, as they could be useful for the mapping of seabed species, such as seagrasses and coral reefs.

After a preliminary assessment of twelve pansharpening techniques, a total of four algorithms was selected for the study. In the literature, four band sensors are mostly selected to carry out this kind of study (Ikonos, GeoEye, QuickBird, etc.); however, we have tried to find the best fused image using an eight-band sensor (WorldView-2).

Both the visual evaluation and the quantitative analysis were achieved using six quality indices at the overall, spectral and spatial level. The best algorithms at the spectral and spatial levels were obtained for each type of ecosystem. Finally, the best fused technique with a compromise between the spectral and spatial quality was identified following the Borda count method. Thus, we provide guidance to users in order to choose the best algorithm that would be more suitable in accordance with the type of ecosystem and the information to be preserved.

It is interesting to observe that, for land regions, the MTF algorithm performs better at preserving the spectral quality, while the weighted wavelet ‘*à trous*’ method through the fractal dimension maps algorithm demonstrates better results considering the spatial detail of the fused imagery. Balancing the spectral and spatial quality, the most appropriate pansharpening algorithm for shrubland and mixed ecosystems is the WAT⊗FRAC technique, while FIHS is selected for the coastal ecosystems. Note that to date, the WAT⊗FRAC algorithm has only been used in agricultural areas; however, we have applied this algorithm in natural vulnerable ecosystems, where a successful result has been obtained. Moreover, we can conclude that the more heterogenic the area to be fused, the smaller the window size in WAT⊗FRAC. FIHS provides the best overall fused image in the simplest scenario. Thus, even though there is no remarkable difference in the way the algorithms perform with respect to land and water areas, we have concluded that images with low variability, such as a coastal scenario, covered mostly by water, require simpler algorithms, rather than more complex and heterogeneous images (i.e., shrubland and mixed ecosystems), which need more advanced algorithms in order to obtain good fused imagery.

Moreover, we have studied the behavior of each algorithm when applied to the complete set of MS bands and on bands covered by and outside of the PAN range. In general, Bands 2–6 mainly have better spatial and spectral quality, but the quality of the remaining bands is acceptable. Analyzing the results, there is a difference in how the same algorithm works on land and coastal areas. The fusion might have higher quality on land, while a lower quality appears in bodies of water.

Additionally, a local study was carried out to identify the distortion introduced in each single band by the best fused algorithms chosen for each scenario. In general, Bands 3–8 attained higher quality for land areas, while in water areas, red and near-infrared bands (5, 7 and 8) experience high spectral distortion. However, these bands are not usually used in seabed mapping applications due to their low penetration capability in water.

Finally, it is important to recall the need to obtain the best fused image in the analyzed ecosystems, as they are heterogenic regions with sparse vegetation mainly made up of small and mixed shrubs with reduced leaf area in the case of shrubland ecosystems and with low radiance absorption in complex and dynamic coastal ecosystems. In this context, thematic maps were obtained using the SVM classifier in the original MS image and in the WAT⊗FRAC fused image. This corroborates the best performance of the WAT⊗FRAC algorithm to generate accurate thematic maps in the shrubland ecosystem. The excellent results provided by these studies are being applied to the generation of challenging thematic maps of coastal and land protected areas, and studies of the state of conservation of natural resources will be performed.

## Figures and Tables

**Table 1:** WorldView-2 sensor technical specifications.

Imaging Mode	Panchromatic	Multispectral
Spatial Resolution	0.46 m	1.84 m
Spectral Range	450–800 nm	400–450 nm (coastal)
		450–510 nm (blue)
		510–580 nm (green)
		585–625 nm (yellow)
		630–690 nm (red)
		705–745 nm (red edge)
		770–895 nm (near IR 1)
		860–1040 nm (near IR 2)

**Table 2:** Location and acquisition date of the three images selected from the Canary Islands.

Worldview-2 Image	Coordinates	Acquisition Date
Teide National Park	28°18′16″ N, 16°33′50″ W	16 May 2011
Maspalomas Natural Reserve	27°44′12″ N, 15°35′52″ W	17 January 2013
Corralejo and Islote de Lobos Natural Park	28°43′52″ N, 13°50′37″ W	28 October 2010

**Table 3:** Indices for the quality assessment of the fused image.

Quality Indices	Equation	Reference	Equation
Spectral Angle Mapper	$\cos^{-1}\frac{\sum_{i=1}^{nband}FUS_{i}\;MS_{i}}{\sqrt{\sum_{i=1}^{nband}FUS_{i}^{2}}\sqrt{\sum_{i=1}^{nband}MS_{i}^{2}}}$	[22]	(1)
Spectral ERGAS	$100\frac{h}{l}\;\sqrt{\frac{1}{nband}\overset{nband}{\underset{i=1}{\sum }}{\left(\frac{rmse_{i}\left(MS\right)}{MS_{i}}\right)}^{2}}$	[23]	(2)
Spatial ERGAS	$100\frac{h}{l}\;\sqrt{\frac{1}{nband}\overset{nband}{\underset{i=1}{\sum }}{\left(\frac{rmse_{i}\left(PAN\right)}{PAN_{i}}\right)}^{2}}$	[24]	(3)
Frequency Comparison	$\frac{1}{nband}\;\overset{nband}{\underset{i=1}{\sum }}corr_{i}\left(dct_{nxn}^{AC}\left(PAN\right),dct_{nxn}^{AC}\left(FUS_{i}\right)\right)$	[25]	(4)
Zhou	$\frac{1}{nband}\overset{nband}{\underset{i=1}{\sum }}corr_{i}\;\left(PAN^{high_{pass}},\;FUS_{i}^{high_{pass}}\right)$	[26]	(5)
Q8	$\overset{nband}{\underset{i=1}{\sum }}\frac{4\sigma_{MS,FUS}\;mean_{MS}\;mean_{FUS}}{\sigma_{MS}^{2}+\;\sigma_{FUS}^{2}\left[{\left(mean_{MS}\right)}^{2}+\;{\left(mean_{FUS}\right)}^{2}\right]}$	[27]	(6)

Note: *nband* is the number of bands; *FUS_i_* represents the fused image; *MS_i_* is the *i*-th band of the MS image; *PAN_i_* is the PAN image; *h* and *l* represent the spatial resolution of the PAN and MS images, respectively; $dct_{nxn}^{AC}$ is the discrete cosine transform computed in blocks of *nxn* pixels, and $corr_{i}$ defines the cross-correlation of the *i*-th band; $FUS_{i}^{high\_pass}$ is the high pass filtered fused image, and $PAN^{high\_pass}$ is the high pass filtered PAN image; σ is the variance of the MS and FUS image.

**Table 4:** Quality results for the complete WV-2 bands and the shrubland ecosystem (best results in bold). SAM: spectral angle mapper; FC: frequency comparison; Spec.: spectral; Spat.: spatial.

	Spectral Quality		Spatial Quality			Global Quality	Borda Count Rank		
	SAM	Spectral ERGAS	Spatial ERGAS	FC	Zhou	Q8	Global	Spec.	Spat.
**FIHS**	3.78	1.68	0.89	0.84	0.72	0.90	13	4	6
**HCS**	**3.52**	0.39	0.91	0.77	0.67	**0.93**	14	**7**	2
**MTF_GLP_HPM**	3.87	**0.33**	0.89	0.81	0.71	0.92	16	6	4
**WAT⊗FRAC**	4.19	1.44	**0.82**	**0.86**	**0.89**	0.90	**17**	3	**8**

**Table 5:** Quality results for the complete WV-2 bands and the coastal ecosystem (best results in bold). SAM: spectral angle mapper; FC: frequency comparison; Spec.: spectral; Spat.: spatial.

	Spectral Quality		Spatial Quality			Global Quality	Borda Count Rank		
	SAM	Spectral ERGAS	Spatial ERGAS	FC	Zhou	Q8	Global	Spec.	Spat.
**FIHS**	1.77	2.91	**2.36**	**0.85**	0.83	0.98	**19**	8	**7**
**HCS**	1.81	1.73	2.64	0.64	0.71	0.98	10	15	2
**MTF_GLP_HPM**	**1.64**	**1.22**	2.61	0.72	0.73	0.98	17	**18**	4
**WAT⊗FRAC**	1.93	2.63	2.54	0.78	**0.88**	0.98	14	15	**7**

**Table 6:** Quality results for the complete WV-2 bands and the mixed ecosystem (best results in bold). SAM: spectral angle mapper; FC: frequency comparison; Spec.: spectral; Spat.: spatial.

	Spectral Quality		Spatial Quality			Global Quality	Borda Count Rank		
	SAM	Spectral ERGAS	Spatial ERGAS	FC	Zhou	Q8	Global	Spec.	Spat.
**FIHS**	7.11	2.98	2.08	0.89	0.73	0.93	12	2	6
**HCS**	5.66	1.73	2.23	0.80	0.61	**0.96**	13	6	2
**MTF_GLP_HPM**	**5.62**	**1.72**	2.23	0.81	0.61	**0.96**	17	**8**	4
**WAT⊗FRAC**	6.88	2.85	**2.05**	**0.93**	**0.98**	0.95	**18**	4	**8**

**Table 7:** Quality results for Bands 1–8 using the best algorithms for each scene. Best results are in bold.

Q8, Block Size: 64	Shrubland Ecosystem	Coastal Ecosystem	Mixed Ecosystem
	Q8 Value for WAT⊗FRAC_w7	Q8 Value for FIHS	Q8 value for WAT⊗FRAC_15
**B1 (Coastal Blue)**	0.696	0.764	0.695
**B2 (Blue)**	0.736	0.889	0.842
**B3 (Green)**	0.878	**0.936**	**0.905**
**B4 (Yellow)**	**0.904**	0.647	0.890
**B5 (Red)**	0.872	0.410	0.851
**B6 (Red Edge)**	0.897	0.395	0.857
**B7 (NIR 1)**	0.841	0.318	0.845
**B8 (NIR 2)**	0.881	0.239	0.832

**Table 8:** Segmentation parameters used for the images and classification accuracy.

Classification Techniques	Support Vector Machine	
Pansharpening Algorithms	Overall Accuracy	Kappa
**MS**	80.61%	0.72
**FIHS**	83.72%	0.76
**HCS**	82.72%	0.75
**MTF_GLP_HPM**	83.18%	0.75
**WAT⊗FRAC**	89.39%	0.85
